# Supplementary material for: The interactome of intact mitochondria by cross-linking mass spectrometry provides evidence for coexisting respiratory supercomplexes
Source: Mol Cell Proteomics. 2017 Dec 8;17(2):216–32. doi: 10.1074/mcp.RA117.000470 (PMC5795388; doi:10.1074/mcp.RA117.000470)
Supplement: Supplemental Data [file supp_RA117.000470_133922_0_supp_23978_fzffwf.zip › spectra_annotation/mito_spectra_annotation/30-1-4-1-8-1.pdf]

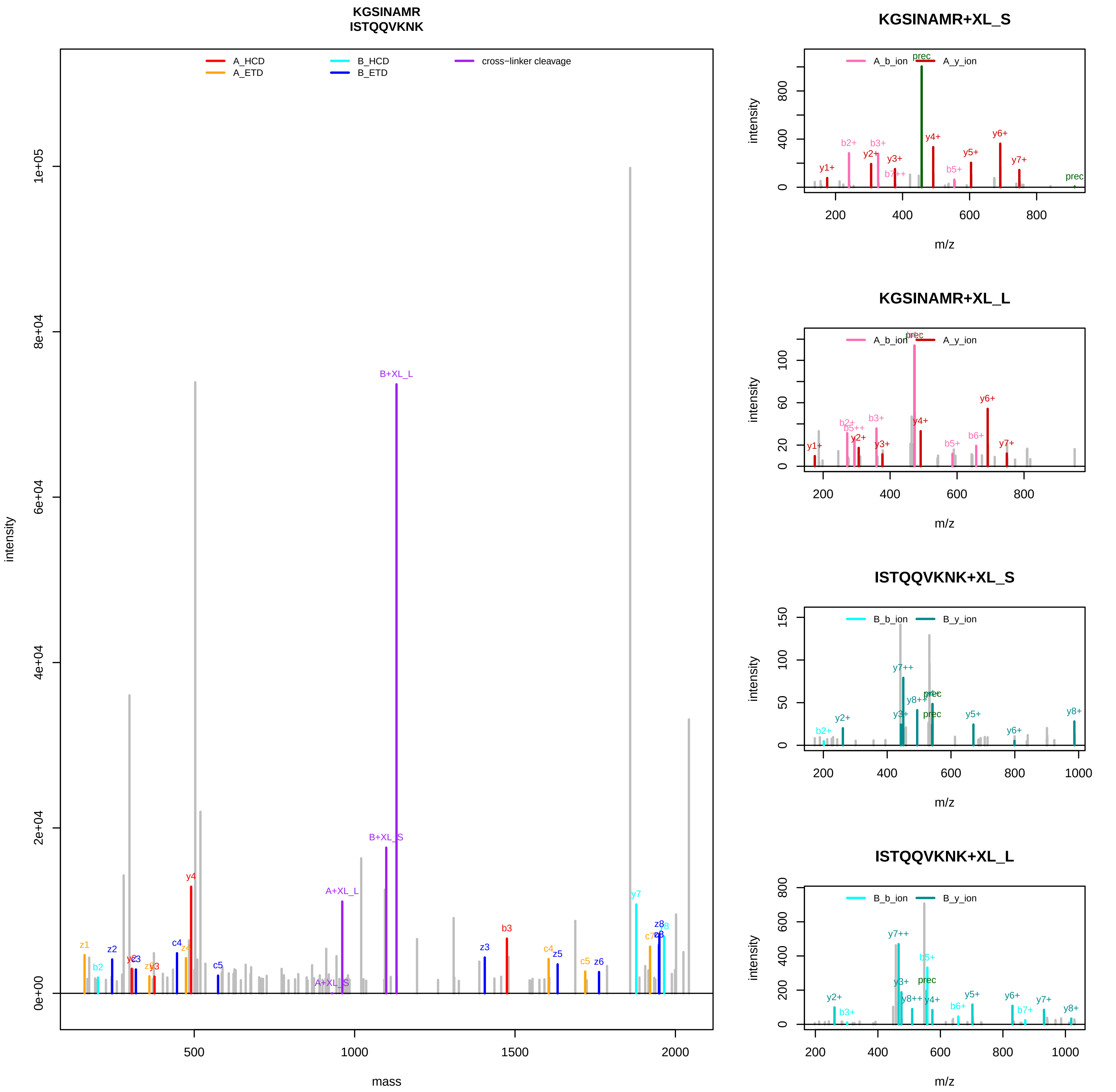
KGSINAMR
ISTQQVKNK
A_HCD
A_ETD
B_HCD
B_ETD
cross−linker cleavage
intensity
mass
0e+00
2e+04
4e+04
6e+04
8e+04
1e+05
500
1000
1500
2000
B+XL_L
B+XL_S
A+XL_L
A+XL_S
y4
b3
y7
z1
b2
z2
c3
y3
c4
z4
c5
z3
c4
z5
c5
z6
c7
z8
KGSINAMR+XL_S
KGSINAMR+XL_L
ISTQQVKNK+XL_S
ISTQQVKNK+XL_L
A_b_ion
A_y_ion
B_b_ion
B_y_ion
prec
m/z
intensity
